# Genome-Wide Characterization and Expression Analysis of Heat Shock Transcription Factors in Two Cultivars of Rice (*Oryza sativa* L.) Under Heat Stress

**DOI:** 10.3390/ijms27167206

**Published:** 2026-08-12

**Authors:** Almas Danish, Muhammad Saeed, Pingfang Yang

**Affiliations:** School of Life Sciences, Hubei University, Wuhan 430026, China

**Keywords:** food security, heat shock factors, climate change, in silico expression, chromosomal localization

## Abstract

Rising temperatures pose daunting challenges for sustainable yield and nutritional quality of rice (*Oryza sativa* L.), thus putting food security at risk. Limited information exists regarding cis-acting regulatory elements and candidate genes controlling the heat shock transcription factor (HSF) gene family in rice. Therefore, the present study identified HSF genes in the japonica (Nipponbare) and indica (9311) rice cultivars through in silico repositories. Three candidate genes (*HSFC2B*, *HSFB1*, and *HSFC2A*) were selected for qRT-PCR analysis to validate their expression patterns under heat stress (HS). The present findings reported a total of 25 *OsHSF* genes through in silico genome-wide identification. Comparative analysis illustrated that the *OsHSF* genes had structural similarities but different expression and transcriptional regulation between the two cultivars. HSF genes were unevenly distributed across the 12 rice chromosomes, suggesting that tandem duplication and gene repetition may have contributed to the evolution of novel genes. Phylogenetic analysis revealed that all *OsHSF* gene family members have shared common ancestry, but several genes lack introns, potentially facilitating swift stress responses as indicated by gene structure analysis. Expression analysis revealed that candidate genes were active, with *HSFC2A* exhibiting the highest level of expression in the japonica cultivar compared to indica under heat-stressed conditions. *HSFC2B* gene showed a higher statistical difference in its response between cultivars, time points, and cultivar vs. time points interactions compared to *HSFC2A* and *HSFB1*. These findings offer valuable insights into the function of *OsHSF* genes that will contribute to the development of climate-resilient rice cultivars.

## 1. Introduction

Rice (*Oryza sativa* L.), a staple food crop, is grown in more than 110 countries, covering an estimated area of 153 million hectares worldwide [1,2]. Rice meets the daily nutritional and dietary needs of over 4 billion people worldwide [3]. The two largest producers of rice, India and China, are situated in tropical and temperate regions and are significantly affected by global warming [4]. Average global temperatures have increased by 1.25 °C and are anticipated to surpass 1.5 °C within the next decade under the current emissions patterns that put both food security and economic development at serious risk [4]. The production of the major staple crops like rice, wheat, soybean, and maize decreases by approximately 3.2%, 6.0%, 3.1%, and 7.4%, respectively, with every 1 °C increase in temperature [5]. Extreme temperatures, whether cold or heat, may severely hinder crops’ development and growth [6]. Rice is highly vulnerable to heat stress (HS) > 35 °C during gametogenesis [7] and flowering [8,9,10]. Heat stress during rice’s reproductive stage, particularly the anthesis, can significantly reduce pollen viability, disturb normal fertilization and pollination, constrain grain development, and ultimately pose a severe threat to rice production [11]. Therefore, there is great urgency to find sustainable solutions to alleviate heat stress-induced impacts on rice. To do so, it is essential to clarify the molecular mechanisms underlying rice response to HS.

Compared to limited exposure to moderately warm temperatures, HS consistently exerts a detrimental effect on rice growth and development, and could lead to cellular damage and even cell death [12]. These effects include hormone imbalance, reduced photosynthesis, damage to the membrane, reactive oxygen species (ROS) accumulation, and slowed carbohydrate metabolism [13]. Consequently, crops are required to develop a variety of intricate and effective mechanisms to sustain normal physiological functions, growth, and metabolic processes under HS. These mechanisms include increasing antioxidant enzymatic activity to scavenge ROS, optimizing photosynthesis, balancing hormones, and adjusting stomatal conductance [14]. Signaling pathways involving nitric oxide (NO), calcium ions (Ca^2+^), ROS, and kinases are key players in heat sensing and signal transduction at the molecular level [15]. Additionally, transcription factors (AP2/ERF, WRKY, NAC, bHLH, MYB) and heat shock factors (HSFs) play a significant role in plant response under HS [16]. Protein located in the plasma membrane, cyclic nucleotide-gated ion channels (CNGC), can also detect the increased membrane fluidity under mild temperature elevation, which facilitates the influx of Ca^2+^ from the periplasm into the cytoplasm [17]. The binding of Ca2+ to the CNGC-associated calmodulins, including AtCaM3, initiates a signaling cascade that results in the phosphorylation of *HSFA1* by a calmodulin-binding protein kinase, like CBK3 [17,18]. Furthermore, the histone variant *H2A.Z* genes ensure that *HSFA1* enables limited access to DNA under HS; thus, the removal of *H2A.Z* enables a significant activation of HS-responsive genes even at normal room temperature [15,19].

Plant heat shock factors have been classified into three groups, A, B, and C, based on their domain structure and sequence homology [20,21]. Under normal environmental conditions, HSFs exist as a monomer in the cytoplasm where they are bound to heat shock protein 70 (HSP70). However, HSFs dissociate from HSP70 and translocate into the nucleus and form trimmers of HSFs that bind to heat shock elements (HSEs) under elevated temperature [22]. Heat shock proteins (HSPs) play a vital part in controlling thermotolerance in plants, the ability of crops to sustain their growth under HS [23]. The oligomerization domain controlling trimerization of HSFs exhibits a bipartite heptad repeat pattern within the hydrophobic-associated region (HR-A/B) [24]. The activation of HSFs involves two main steps: (**a**) the exposure of one or more specific activator domains, and (**b**) the induction of high-affinity binding to heat shock promoters, which is facilitated through trimerization and the formation of synergistic interactions among the HSF trimer [25]. Heat shock proteins are synthesized when HSFs bind to HSEs, located in the promoter region of the HS-responsive genes, which initiates transcription leading to the production of HSPs under HS [26]. These proteins build a complex regulatory network that governs and controls the expression of genes associated with HS [27]. These HSPs, which function as molecular chaperones, are responsible for regulating the accumulation, localization, destruction, and folding of unfolded proteins in crops [28].

In Asia, rice has two primary subspecies (japonica and indica) after going through the extensive process of selection and domestication, which are shaped by adaptability to climatic conditions and geographical locations [29]. Under adverse conditions, both species illustrate distinct physiological, morphological, biochemical, and molecular traits, leading to distinct performances [30]. For instance, japonica species exhibits low heat tolerance related to indica rice [30]. This highlights the significance of elucidating the inherited basis controlling the variances between japonica and indica, which can be substantial for breeding programs designed to develop heat-tolerant rice [31]. Therefore, it is critical to gain an understanding of how japonica and indica respond to elevated temperatures for generating holistic approaches to improve general heat tolerance in rice.

Previous research analyzed the *OsHSFs* gene family in rice but mainly relied on in silico investigation through transcript data profiling [32,33]. Genome-wide in silico investigation revealed that 25 *OsHSFs* genes were expressed in rice [34]. In the Nipponbare cultivar, expression of class A Os*HSFs* genes was higher than class B and class C [33]. Studies have investigated corresponding HSFs expression analysis between japonica and indica cultivars, but no domain analysis and subcellular localization were measured. Although the HSF gene family has been characterized in rice, limited information is available on the comparative expression patterns and potential regulatory roles of HSF genes in heat-tolerant and heat-sensitive cultivars under heat stress conditions. Furthermore, no research has investigated the cis-acting elements and molecular mechanisms of the HSF gene family in the rice genome under HS. Therefore, the contemporary study was designed to bridge these research gaps and provide a comprehensive analysis for the identification of potential candidate *OsHSFs* genes. The primary objectives of the present study are to: (**a**) perform a genome-wide characterization of the HSF gene family, (**b**) compare the expression profiles of *OsHSFs* genes in contrasting rice cultivars under heat stress, and (**c**) identify candidate *OsHSF* genes potentially associated with heat tolerance. This study will contribute significantly by providing valuable insights into the thermotolerance mechanism of rice, potentially contributing to the development of resilient rice cultivars.

## 2. Results and Discussion

### 2.1. Identification of the OsHSF Gene Family and Its Chromosomal Location

The present study identified a set of 25 genes associated with HS in both cultivars, Nipponbare and 9311. The findings were similar to previous research, where 25 unique *OsHSF*-related genes were identified in the Nipponbare cultivar after excluding duplicate sequences [34,35]. By accessing the MSU Rice Genome Annotation Project database, comprehensive details were collected, including gene names, alternative splice variants, genomic length, coding sequence/conserved domain sequence (CDS) length, protein length, and the potential functions of these *OsHSFs* genes (Table 1). Furthermore, the chromosomal distribution of identified *OsHSFs* genes was comprehensively elaborated using the extensive genomic data of the rice varieties Nipponbare and 9311 (Table 2).

**Figure 1:**
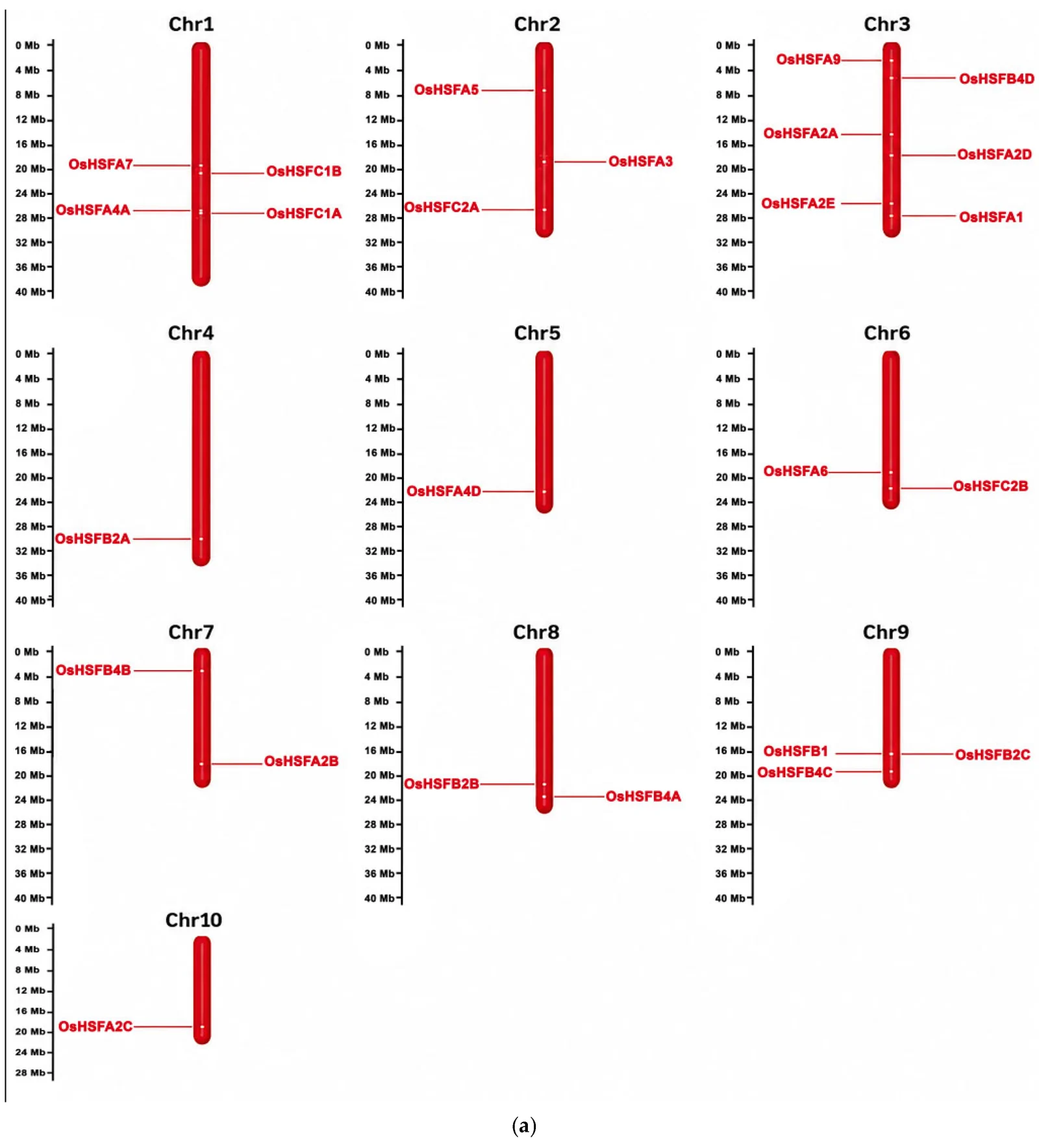

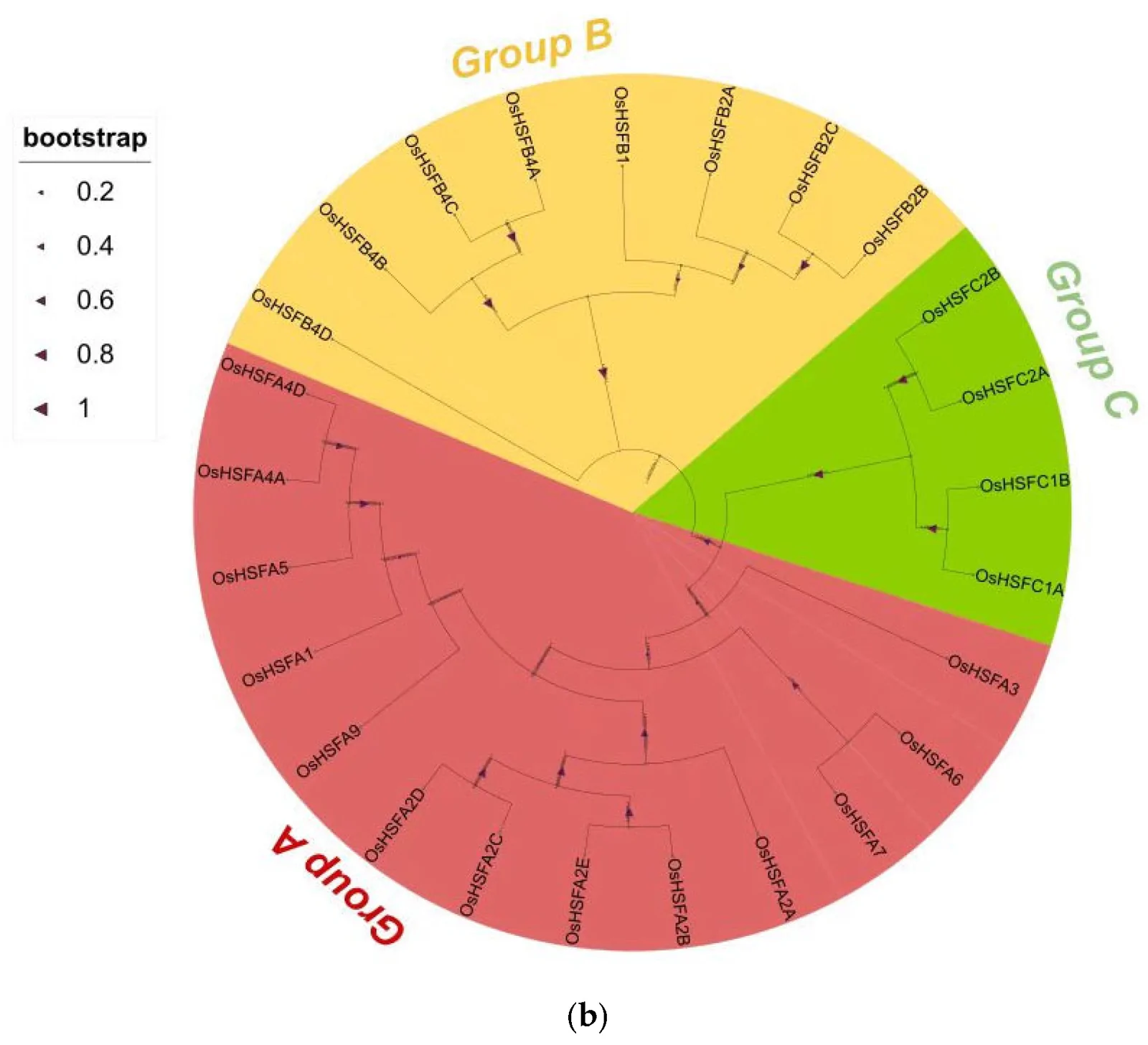
(**a**) Chromosomal distribution of the *OsHSF* genes on the rice chromosomes. Gene positions are indicated by red labels, while orange bars represent individual chromosomes. (**b**) Rooted neighbor-joining phylogenetic tree of the *OsHSF* gene family. The tree classifies *OsHSF* proteins A, B, and C, with bootstrap values indicating branch support.

The physical positions of the HSF genes were determined in megabases (Mbs) to establish their chromosomal locations with precision (Figure 1a). Chromosomal mapping showed a non-uniform distribution pattern across the rice genome, as indicated by the chromosomal distribution analysis of the *OsHSF gene* [32,35]. The *OsHSF* genes were discovered to be most abundant on chromosome 3 with 6 genes (*OsHSFA9*, *OsHSFB4D*, *OsHSFA2A*, *OsHSFA2D*, *OsHSF2E* and *OsHSFA1*) and chromosome 1 with 4 genes (*OsHSFA7*, *OsHSFC1B*, *OsHSFA4A*, *OsHSFC1A*). However, most *OsHSF* genes were mapped to the shorter arm of their respective chromosomes, except for *OsHSFC2A*, *OsHSFA2D*, *OsHSFA9*, *OsHSFB4D*, and *OsHSFA2B*. In contrast, chromosomes 4 and 5 each carried a single copy of an *OsHSF* gene, *OsHSFB2A* and *OsHSFA4D*, respectively, while chromosomes 6, 7, and 8 carried two paralogous *OsHSF* genes, and chromosomes 2 and 9 each carried three paralogous genes (Figure 1). The location of *OsHSFs* genes in the genomes of 9311 and Nipponbare rice cultivars indicated a complex genetic structure that could be a possible explanation for rice’s response to HS [36]. Identification of various *OsHSFs* genes on different chromosomes suggested that rice’s reaction to HS may be diverse and redundant. Such genetic diversity could be the reason behind the different tolerance potential of rice cultivars towards HS [37].

### 2.2. Genetic Relationship Between OsHSFs Genes

The circular rooted phylogenetic tree exhibited the genetic relationship among different *OsHSFs* in both rice cultivars (Figure 1b). The branches of the phylogenetic tree represented the evolutionary divergence, with each branch indicating a duplication event or genetic variation [38]. The phylogenetic tree is based on sequence homology, which suggests that H shares a common ancestor [39]. The tree is categorized into 3 chief clusters (group A, group B, and group C), where group A shares a large number of genes, each represented by a different colour. This was in accordance with previous research conducted in potato, tomato, peanut, and rice cultivar Nipponbare [32,40,41,42]. Group A is further subdivided into subgroups with a total of 13 genes, including (OsHSF*A1*, *OsHSFA2A*, *OsHSFA2B*, *OsHSFA2C*, *OsHSFA2D*, *OsHSFA2E*, *OsHSFA3*, *OsHSFA4A*, *OsHSFA4D*, *OsHSFA5*, *OsHSFA6*, *OsHSFA7*, *OsHSFA9*). Such genes were grouped because it is possible that they share a more recent common ancestor and may have undergone gene duplication, a similar regulatory mechanism, and function [38]. Group B is further classified into subgroups containing genes (*OsHSFB1*, *OsHSFB2A*, *OsHSFB2B*, *OsHSFB2C*, *OsHSFB4A*, *OsHSFB4B*, *OsHSFB4C*, and *OsHSFB4D*). Moreover, group C comprises genes like *OsHSFC1A*, *OsHSFC1B*, *OsHSFC2A*, and *OsHSFC2B*. Understanding these relationships between genes in one group and with other groups can assist future studies in elucidating the roles of specific HSF genes during HS (Figure 1b).

### 2.3. Structure of HSF Family Genes

**Figure 2:**
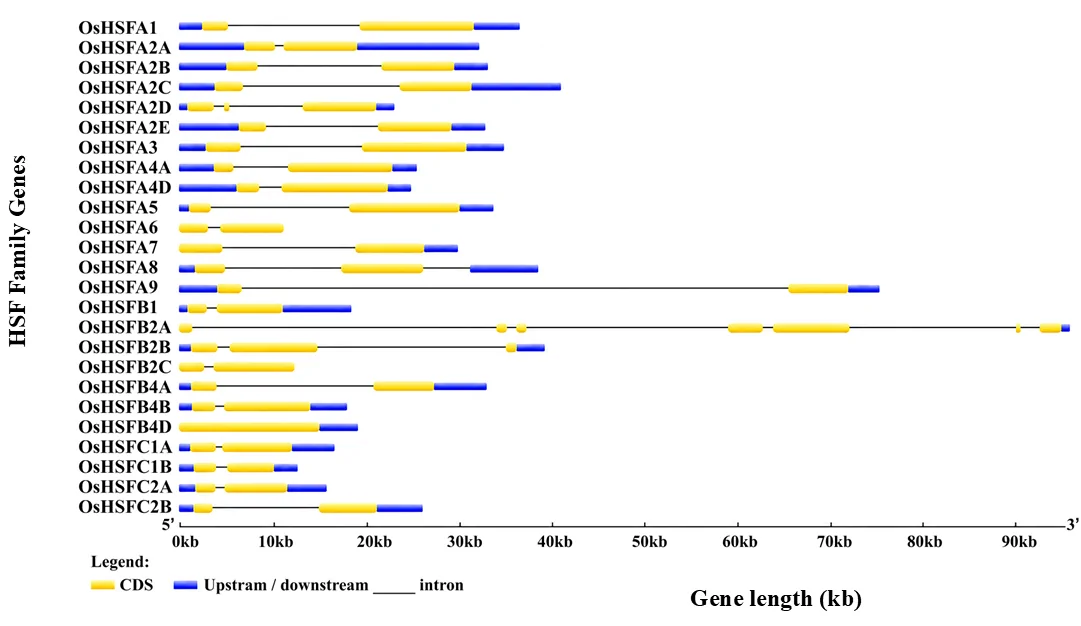
Gene structure analysis of the *OsHSF* gene family showing the distribution of CDSs (yellow), UTRs (blue), and introns (black lines).

A schematic representation of the intron-exon architecture of the *HSF* genes was illustrated in Figure 2. Gene structure, with exons-portions of the gene encoding protein sequences (blue colour) and introns-non-coding intervening sequences (black line). The *x*-axis quantified gene length in base pairs (bps), while the *y*-axis enumerated the distinct *HSF* genes, categorized by family (e.g., HSFA, HSFB) [43] and annotated with specific gene identifiers (e.g., LOC_Os02g48770). Notably, intron-less genes may facilitate swift responses to stress and contribute to the regulation of crop growth and development under stress [44]. The OsHSF genes showed significant differences in the organization of their structures, especially in the number and size of introns, with more similar members showing more similar structures [45]. This variation is caused by the evolutionary history of the gene family, and it is inferred that additional diversification of the family has occurred by intron gain and loss after the gene duplications. The structure of the genes may account for differences in gene expression, alternative splicing, and transcriptional regulation, causing divergence without compromising the overall function of HSF proteins in plant growth, development, and responses to stress [46] (Figure 2). This figure displays the gene structure of OsHSF family genes of rice, which are divided into three subfamilies, Hsfa, Hsfb, and Hsfc. The genes are shown from the 5′-end to the 3′-end, where the yellow blocks indicate coding sequences (CDS/exons), the blue blocks indicate upstream and downstream UTR regions, and the thin black lines indicate introns. The genomic structure of most genes is simple, consisting of 1–2 exons and very short introns, a typical genomic organization of *HSF* genes. A few genes have unusually long introns or long gene lengths, such as OsHSFB1 (~7 kb) and especially OsHSFB2B, which is about 9–10 kb in length, consisting of multiple small exons within a long intron [47]. A striking similarity in intron-exon patterns was observed among OsHSF genes within the same class and subclass for intron phase, intron number, exon length, and whole gene length. This conservation of gene structure within specific *OsHSF* classes and subclasses may reflect functional constraints and shared ancestry, underscoring the importance of genes in the HS response of rice [48].

### 2.4. Promoter Sequence Analysis of the OsHSF Family Genes

**Figure 3:**
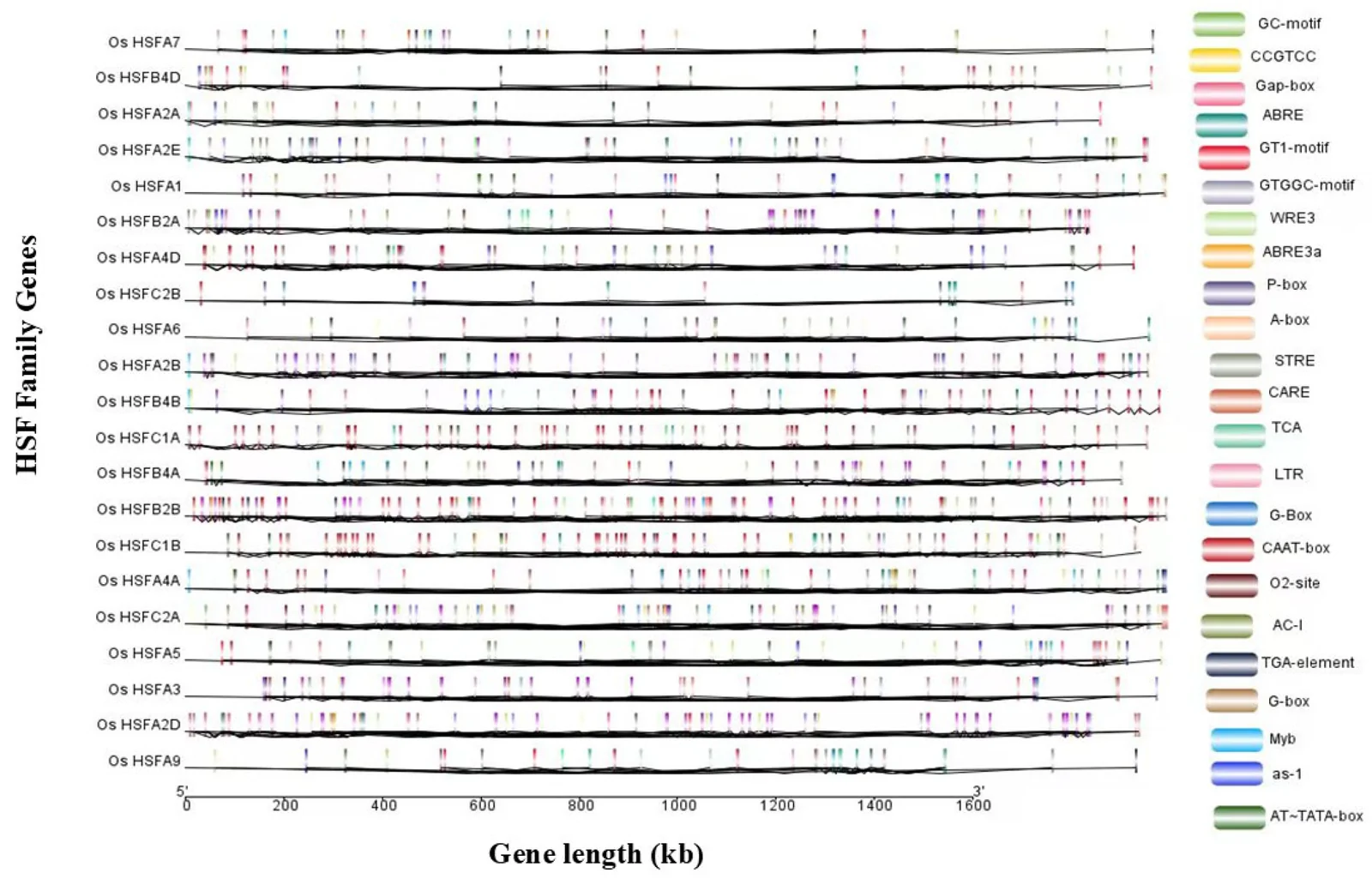
Cis-acting regulatory elements identified in the promoter regions of *OsHSF* genes. Different colors indicate various stress-, hormone-, light-, and development-related cis elements.

Ensemble Plant and PLANT CARE 1500 bp upstream area of each gene identified a promoter sequence of *OsHSF* and cis-regulatory elements in Nipponbare and 9311 (Figure 3). In the current study, a total of 23 different cis elements were found across OsHSFs genes (Figure 3). The length of the bar indicated the cis elements’ span within the sequence. Furthermore, each gene’s sequence length varied from 300 to 1500 bps. Heat transcription factors (TFs) bind to cis-regulatory elements on the promoter region of stressed genes to activate and deactivate genes involved in a particular biological activity [49]. Rice can use a small number of TFs to regulate gene expression under various environmental stimuli through synergistic coordination of multiple TFs [50,51,52,53]. The promoter region’s cis-acting elements of the rice genome might be responsible for monitoring the expression of genes under stress conditions [33,35]. The cis-regulatory elements were observed in their respective places, such as Gap-box, which might be an essential component for crops to adjust their development and growth in response to light [54]. MYB TFs play a crucial role in stress response, development, and secondary metabolism [54]. Furthermore, ABA-responsive elements (ABRE) might be a crucial cis-acting regulatory element in crops, regulating gene expression in response to the hormone abscisic acid (ABA), which is necessary for crops to alleviate environmental stresses like extreme temperatures, high salinity, and drought [55]. On the other hand, the anti-Repression Element (ARE) is crucial for controlling the expression of certain genes. The present study identified MYB, ABRE, and ARE cis-regulatory elements across OsHSFs genes, enhancers, and TFs (Figure 3) that play a vital function in controlling the genes’ expression, frequently traveling great distances to physically approach the promoters of the genes that they target [56]. CCGTCC, AE-box, TCA, AT-TATA-box, A-box, and CAAT-box may be involved in hormonal signaling and stress responses [54]. Additional experimental research is required to clarify the functional significance of these regulatory elements (ABRE and ARE) that might be linked to ABA signaling, thus indicating a crosstalk between HS and hormonal signaling pathways. Such information is crucial for comprehending the underlying molecular mechanisms controlling HS responses in rice cultivars [57].

### 2.5. Motifs and Conserved Motifs Analyses of OsHSFs

**Figure 4:**
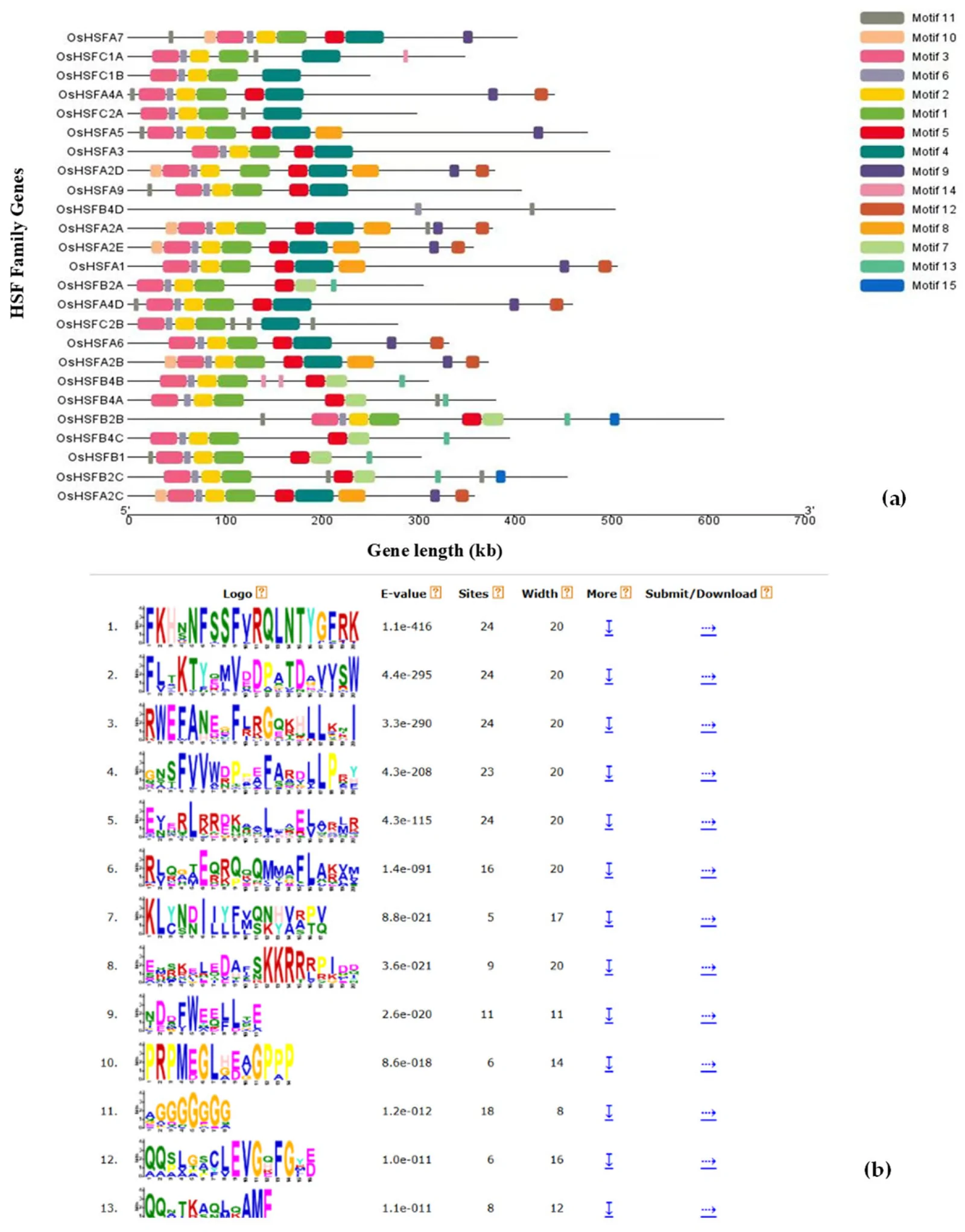
Distribution of (**a**) motifs; and (**b**) conserved motifs on the promoter region in the *OsHSFs* gene family. Distribution of conserved motifs (Motifs 1–15, color-coded) across *OsHSF* protein sequences, arranged by phylogenetic clustering.

The motifs, which are short, recurring patterns in DNA, are associated with mitochondria and chloroplasts [32]. The motifs and conserved motifs analyses are very important for understanding the transcriptional regulation mechanism of HSFs, and act in the rice response to HS. These analyses also revealed that the multiple arrangements of motifs likely contribute to the distinct functionalities exhibited by each group [58]. Such motifs have a critical biological function in the HSFs of both rice cultivars under stress (Figure 4). In this stacked bar chart, each row corresponds to a distinct *OsHSF* gene identified by its locus of confidence (LOC) number. Previously, similar findings were reported for motifs for HSFs in *Camellia sinesis*, *Cucurbita moschata*, and *Phyllostachys edulis* [41,59,60]. Our research predicted 15 conserved motifs related to *OsHSFs* genes in both rice cultivars in the promoter region (Figure 4). Similarly, 15 conserved motifs related to *OsHSFs* were previously reported [32]. The presence of these motifs can indicate the existence of a complex regulatory system that controls rice responses under HS stress. The conserved motif analysis revealed that the majority of *OsHSF* proteins contained highly conserved motifs similar to the DNA-binding domain (DBD) and the HR-A/B oligomerization domain, which indicated their critical function in DNA binding and stress-induced transcription. Functional diversification and evolutionary divergence after gene duplication can be seen from differences in the motifs of the members in *OsHSF*. The presence of these motifs in the conserved regions implies that they are highly selectively filtered and that they are important in maintaining biological functions. However, the absence or presence of certain motifs can give rise to specific functions in regulation, allowing the *OsHSF* proteins to play a role in responding to different environmental stresses and developmental processes. For instance, HSEs can bind to their promoter region, that indicated a potential auto-regulatory mechanism of HSF [32]. Furthermore, the motif also contained many known regulatory elements such as TATA-box, CCAAT-box, AGAAA-motif, GC-motif, WRE3, ABRE4, CCGTCC, ACGT1, ACE, CCGTCG, TGA-elements, and Sn1 and others (Figure 4).

### 2.6. Conserved Domain Analysis

**Figure 5:**
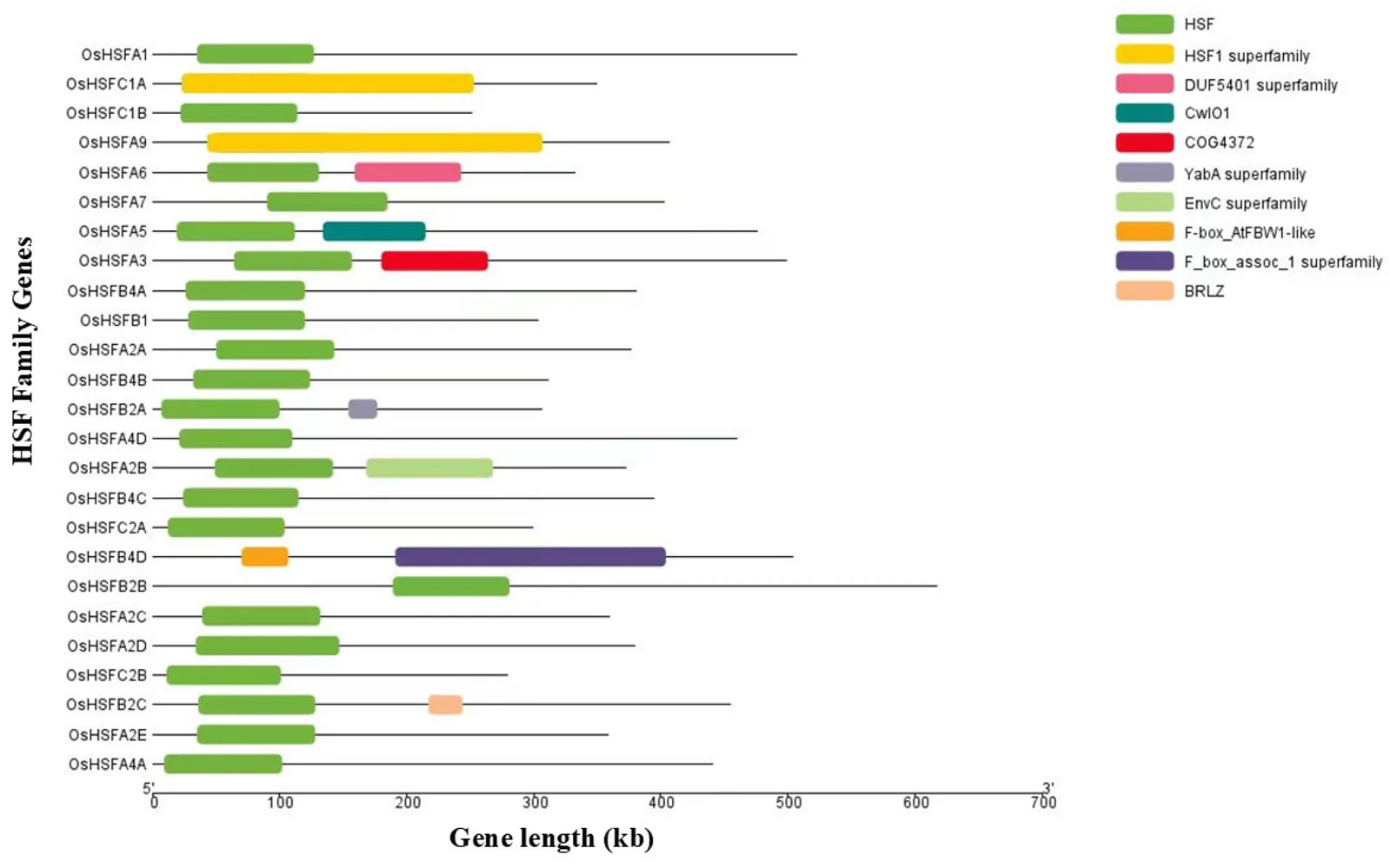
Conserved domain organization of the *OsHSF* proteins. Colored boxes indicate the HSF domain and other conserved superfamilies identified by domain analysis, while black lines represent protein lengths.

Our findings indicated the presence of a conserved domain in both rice cultivars, characterized by sequence patterns of conserved motifs or a self-folding unit of protein (Figure 5). These features may facilitate their identification in polypeptide sequences in the motif on the promoter region [49,61]. The present analysis illustrated the distribution and composition of conserved domains across the promoter region of *OsHSF* genes in rice, illustrated by brighter colored bars (Figure 5). The intensity of the red shade correlated with the degree of conservation of residues, which may play a role in regulating HS responses. Our findings were supported by the previous literature, which observed a similar trend regarding the conserved domain across HSF [38,49]. Moreover, the diversity of families and superfamilies, including *HSF1*, may play a key role as a major TF in regulating the HS response of genes, which may occur in response to cellular stresses like oxidative damage, temperature fluctuations, and misfolded proteins [62]. HSF can bind to DNA on the promoter region by the conserved domain architecture of *OsHSF* proteins, with the core HSF domain (green) present near the N-terminus in all sequences, confirming their identity as heat shock factors, while a subset of proteins (e.g., *OsHSFC1A*, *OsHSFA9*, *OsHSFA6*, *OsHSFA5*, *OsHSFA3*, *OsHSFB2A*, *OsHSFA2B*, *OsHSFB4D*, *OsHSFB2C)* carry additional secondary domains such as *HSF1* superfamily, *DUF5401*, *CwlO1*, *COG4372*, *YabA*, *EnvC*, *F-box_AtFBW1-like*, *F_box_assoc_1*, *and BRLZ*, most of which are likely low-confidence generic domain-database hits rather than biologically characteristic HSF motifs [63]. Similarly, the presence of conserved domains such as the HSF_DNA-bind superfamily suggested the binding and functions of these *OsHSFs* [33].

### 2.7. In Silico Gene Expression Study of Nipponbare and 9311 Cultivars

**Figure 6:**
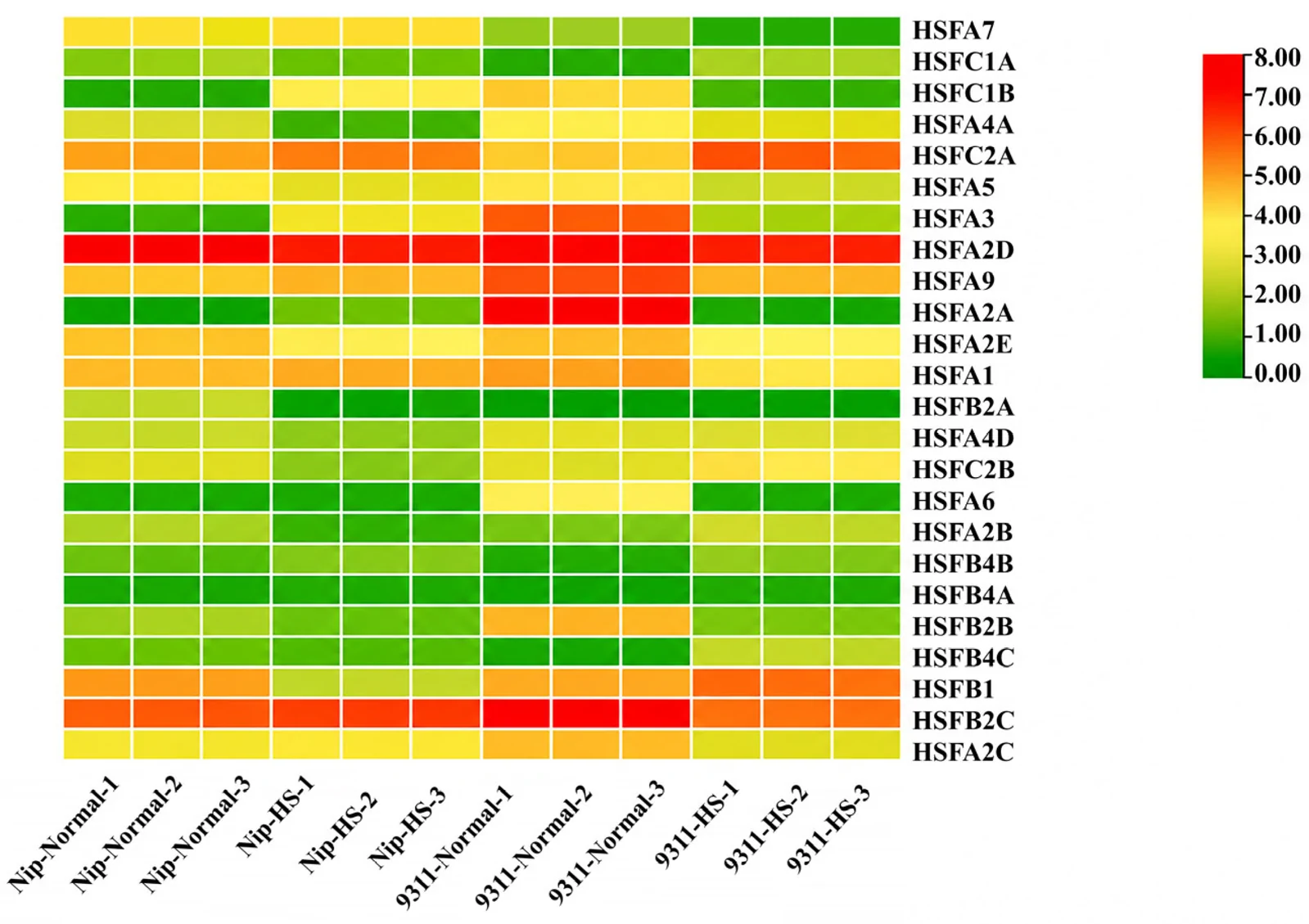
Heatmap of *OsHSFs* gene expression in Nipponbare (*japonica*) and 9311 (*indica*) rice under normal and heat stress (HS) conditions. NIP (Nipponbare), NiP Normal-1 (NIP cultivar, biological replicate 1, control), NiP Normal-2 (NIP cultivar, biological replicate 2, control), NiP Normal-3 (NIP cultivar, biological replicate 3, control), NiP-HS-1 (NIP cultivar, biological replicate 1, heat stress at 36 h), NiP-HS-2 (NIP cultivar, biological replicate 2, heat stress at 36 h), and NiP-HS-3 (NIP cultivar, biological replicate 3, heat stress at 36 h) and same replicates under control and HS for cultivar 9311.

Utilizing the transcriptomic data, we constructed a heat map to illustrate gene expression profiles for both Nipponbare (japonica) and 9311 (indica) cultivars under control and HS conditions (Figure 6). A subset of genes exhibited both downregulation and upregulation across the two subspecies of rice, with distinct temporal dynamics [64]. For example, the *HSFA5* gene demonstrated a gradual decline from Nip-normal-1 to Nip-normal-3 in japonica, whereas in indica, its downregulation commenced later at 9311-normal-2. Under HS conditions, *HSFB2B* is rapidly suppressed in Nip-HS-1 in japonica, continuing through Nip-HS-3, while in indica, the decrease initiated at 9311-HS-2. The *HSFA3* gene presented an intriguing case, exhibiting an increasing expression trend in japonica from Nip-normal-1 to Nip-HS-3, in contrast to a decreasing trend in indica from 9311-normal-1 to 9311-HS-3. Gene *HSFA6* exhibited stable expression levels across diverse conditions and subspecies because *HSFA6* demonstrated low expression throughout all developmental stages in japonica and a diminishing response in indica from 9311-HS-1 to 9311-HS-3, indicating a strong evolutionary conservation of its regulatory mechanisms. Additionally, *HSFB2B* displayed a similar response to *HSFA6* in both subspecies (Figure 6). Our analysis also showed that the *OsHSF* gene family is highly conserved between the japonica cultivar Nipponbare and the indica cultivar 9311, with both genomes containing the same three major HSF classes (A, B, and C) and exhibiting similar conserved domains, motif compositions, and exon–intron organizations. This high level of structural conservation suggested that the *OsHSF* family has been subjected to strong evolutionary constraints to preserve its essential regulatory functions in rice.

*HSFC2B* expression level increased in the cultivar 9311 but declined in Nipponbare, which caused the regulation of heat-responsive genes to occur sooner in the cultivar 9311 compared to the Nipponbare cultivar. Conversely, *HSFC2A* showed an upward trend in both cultivars across all stages (Figure 6). Genes with increasing trends usually have perfect HSE repeats with ideal spacing (17–20 bps), particularly in the japonica variants [65]. On the other hand, genes with decreased expression usually contain degenerate HSE motifs or repressive chromatin marks, with more pronounced silencing mechanisms in indica species [65]. Genes with stable expression patterns have highly conserved promoter regions among subspecies, which accounts for their consistent expression patterns. These observations suggest that japonica adopts a rapid-response strategy with early gene activation, while indica follows a more gradual yet sustained response approach [31]. The distinct regulation of *HSFA7*, *HSFC1B*, and *HSFA2A* compared to *HSFC2B* and *HSFB1* suggested subspecies-specific adaptation to HS conditions. The conserved regulation of *HSFC2A* underscored its crucial role in the stress response across rice cultivars. Our analysis illustrated how variations in temporal regulation and promoter architecture contribute to stress adaptation strategies in rice cultivars. The expression patterns specific to stages and subspecies offer valuable insights for developing climate-resilient rice cultivars by targeting *OsHSF* gene regulatory networks [66]. Despite overall conservation, differences were reported in the transcriptional responses of several *OsHSF* genes between the indica and japonica cultivars, highlighting functional variations at the regulatory level rather than the structural level. Expression profiling showed cultivar-dependent patterns for *OsHSF* genes like *HSFC2B*, *HSFC2A*, *HSFA3*, *HSFA2D*, and *HSFB1* between the two cultivars (Figure 6). For instance, the *HSFB1* gene showed higher expression under HS compared to the control in cultivar 9311. However, the Nipponbare cultivar showed the opposite trend for this gene under HS stress compared to control (Figure 6).

### 2.8. Gene Expression of OsHSFs

**Figure 7:**
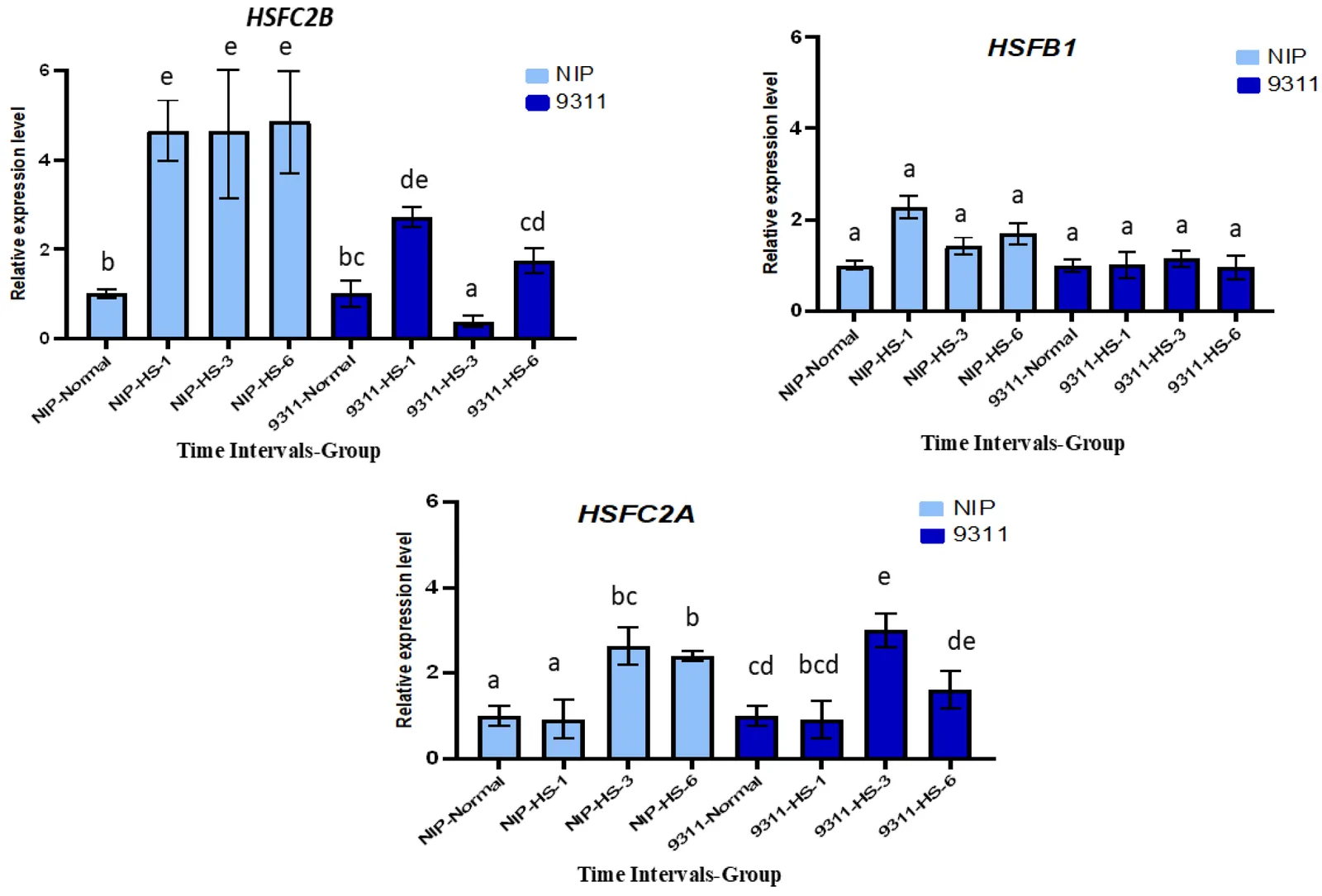
Relative expression of *HSFC2B*, *HSFB1*, and *HSFC2A* determined by qRT-PCR at 0 (normal), 1, 3, and 6 h after heat stress. Relative expression was calculated using the 2^−ΔΔCt^ method and is presented as mean ± SD (n = 3). Statistical analyses were performed on ΔCt values using two-way ANOVA followed by Tukey’s multiple comparison test (*p* < 0.05). Different lowercase letters indicate significant differences among time points between Nipponbare and 9311. *X*-axis shows cultivar + time intervals (h) group and *y*-axis represent relative expression levels.

In the Nipponbare cultivar, the expression of the *HSFC2B* was at its lowest at the 0 h time point, which served as the baseline for comparison (Figure 7). A significant increase in expression was observed at both 1 and 3 h post-treatment, with the levels being almost the same in both time points. The *HSFC2B* gene in Nipponbare indicated a swift and strong reaction to HS, with its expression level further rising even more at the 6 h mark, suggesting a continued increase under HS. Conversely, the 9311 cultivar exhibited a non-linear pattern in the expression of the *HSFC2B* gene. The maximum expression was observed at the 1 h time point, and the lowest was measured at the 3 h time point. At the 6 h time point, the expression level was moderate, suggesting a partial recovery or a secondary response to HS. Furthermore, two-way ANOVA also exhibited significant differences between cultivars (*p* = 5.83 × 10^−8^), time points (*p* = 1.28 × 10^−8^), and cultivar x time points (*p* = 7.71 × 10^−8^) (Table A2). The differential expression patterns of the *HSFC2B* gene between the two cultivars suggested distinct mechanisms of HS response [36,67]. The Nipponbare cultivar exhibited a biphasic response with high expression at the 6 h point compared to the 0 h point, which suggested the activation of multiple HS response pathways or the involvement of feedback mechanisms that sustained the *HSFC2B* expression over time [67]. On the other hand, a non-linear pattern in cultivar 9311 indicated the activation of negative feedback pathways that decrease the *HSFC2B* expression after an initial increase in its expression under HS [68]. Furthermore, the involvement of other HSFs in cultivar 9311 could be responsible for the stable expression of *HSFC2B* at the 6 h time point post-treatment. Such distinct patterns of *HSFC2B* in both cultivars underscored the complexity of rice genetic makeup and responses under HS [69]. The continuous upregulation of *HSFC2B* in the Nipponbare cultivar might contribute to its greater HS tolerance as compared to 9311 (Figure 7).

The expression of the *HSFC2A* gene was lowest at the 1 h point and maximum at the 3 h point for both cultivars (Figure 7). At the 6 h point, the Nipponbare cultivar showed significantly higher expression compared to 9311. Such a distinct expression of the *HSFC2A* gene between the two cultivars indicated a conserved mechanism of HS response [67]. The significant increase in *HSFC2A* gene expression at the 3 h point, with slightly low but stable expression at the 6 h point in Nipponbare, indicated that activation of HS-responsive pathways to alleviate stress [67]. The overall gene expression in response to HS was less pronounced in cultivar 9311 as compared to Nipponbare, indicating that the regulation of gene expression in cultivar 9311 may be less sensitive to HS or that other *OsHSFs* might exert a more predominant influence on its HS response [2]. Furthermore, two-way ANOVA also exhibited significant differences between cultivars (*p* = 5.01 × 10^−11^) and time points (*p* = 1.22 × 10^−6^), but a non-significant difference for cultivar x time points (*p* = 0.326) (Table A2). The differences in genetic expression of these candidate genes could be a potential point of interest for breeders to develop heat-resilient rice cultivars. The expression of the *HSFB1* gene was lowest at the 0 h point in the Nipponbare cultivar, while maximum expression was observed at the 1 h time point (Figure 7). However, this cultivar showed a decrease in its expression at the 3 h point, but a gradual increase at the 6 h point, which indicated a sustained yet decreased response compared to the 1 h point. On the other hand, cultivar 9311 showed a distinct pattern of the *HSFB1* gene, with maximum expression observed at the 3 h point. This cultivar did not show a significant difference in its expression between various time points (Figure 7). Furthermore, two-way ANOVA exhibited non-significant difference between cultivars (*p* = 0.183), time points (*p* = 0.327), and cultivar x time points (*p* = 0.283) (Table A2). The overall low expression of the *HSFB1* gene in cultivar 9311 compared to Nipponbare showed its low tolerance to HS or less efficient regulatory mechanisms to alleviate stress. On the other hand, Nipponbare might activate multiple HS-responsive pathways to combat stress [61].

## 3. Materials and Methods

### 3.1. Identification of OsHSF Genes in the Genome of Rice

Heat Shock Transcription Factor family genes were identified in two rice cultivars, Japonica (Nipponbare) and Indica (9311), using the BLAST (SequenceServer v3.1.3) search tool provided by the Rice Annotation Project Database (RAP-DB v1.0). The genomes of Nipponbare and 9311 were first mined using MSU ID for *OsHSF* genes at the MSU Rice Genome Annotation Project Database, the Database of Rice Transcription Factors (DRTF), the National Center for Biotechnology Information (NCBI), and the Plant Genome Database (PlantGDB) [32].

### 3.2. Chromosomal Localization

MapGene2Chrom (http://mg2c.iask.in/mg2c_v2.1/) (accessed on 10 November 2025) was employed to generate genes’ chromosomal localization.

### 3.3. Phylogenetic Tree

To eliminate redundant sequences, the protein sequences obtained from multiple public repositories were associated using Clustal Omega v11.0 [32]. A combined rooted neighbor-joining (NJ) polygenetic tree of both varieties was developed with bootstrap (1000 replicates) and the default parameters (pairwise deletion) using the MEGA11.0 software.

### 3.4. MEME Motif and Conserved Domain Analyses

Heat shock factor motifs protein sequences from both rice cultivars were identified utilizing the Multiple Em for Motif Elicitation online tool (MEME Suite version 5.5.0) (https://meme-suite.org/meme/tools/meme) (accessed on 25 October 2025). Conserved domains in both rice varieties were identified using the NCBI (NIH) web CD search tool CDD v3.21 (https://www.ncbi.nlm.nih.gov/) (accessed on 10 November 2025) and TBtool [32].

### 3.5. Structure of OsHSF Family Genes

By using the Gene Structure Display Server (GSDS) (https://gsds.gao-lab.org) (accessed on 20 April 2025), full-length cDNA transcripts were aligned with genomic sequences and the locations of introns and exons in *OsHSF* genes [70]. For a single full-length cDNA that was matched to a continuous segment of genomic sequence, the introns are the spaces between exons that are entirely composed of genomic sequence, while exons are the closest homologous sequence blocks present in both genomic and full-length cDNA sequences [71]. The lengths of each exon are added to determine the overall length of a gene to understand the size and range of *OsHSF* family genes.

### 3.6. Promoter Sequence Analysis of OsHSF Family Genes

Each gene’s promoter region was found using the EnsemblPlants (v61) database (http://plants.ensembl.org/index.html) (accessed on 10 July 2025). A cis-regulatory element analysis of the *OsHSF* gene family in promoters was performed using a 1500 bp upstream area of each gene by PLANT CARE (https://bioinformatics.psb.ugent.be/webtools/plantcare/html) (accessed on 12 July 2025) and TBtool v2.503 [32].

### 3.7. In Silico Gene Expression

Transcriptomic data were obtained from the Gene Expression Omnibus (GEO) database under accession GSE144566 (https://www.ncbi.nlm.nih.gov/geo/query/acc.cgi?acc=GSE144566) (accessed on 20 November 2025) to validate the expression of *OsHSF* genes in the rice cultivars. Using the TBtool softwarev2.503, a heatmap was created from this information. Furthermore, cross-referencing the expression profiles of the candidate genes was done using the Rice Expression Profile database (RiceXPro) [32]. Three potential candidate genes were selected for additional authentication using the quantitative reverse transcriptase polymerase chain reaction (qRT-PCR).

### 3.8. Experimental Conditions

Seeds of Nipponbare (NIP) and 9311 were germinated and grown in a growth chamber under precise conditions (12 h photoperiod, 28 °C/25 °C day/night temperature, 70% relative humidity) [72]. After two weeks, seedlings were selected and subjected to heat stress in a growth chamber set at 42 °C. Leaf samples were collected at designated time points: 0 h (control, CK), 1 h, 3 h, and 6 h after the start of treatment. All samples were immediately frozen in liquid nitrogen and preserved at −80 °C until further processing.

### 3.9. Quantitative Real-Time PCR Analysis

**Figure A1:**
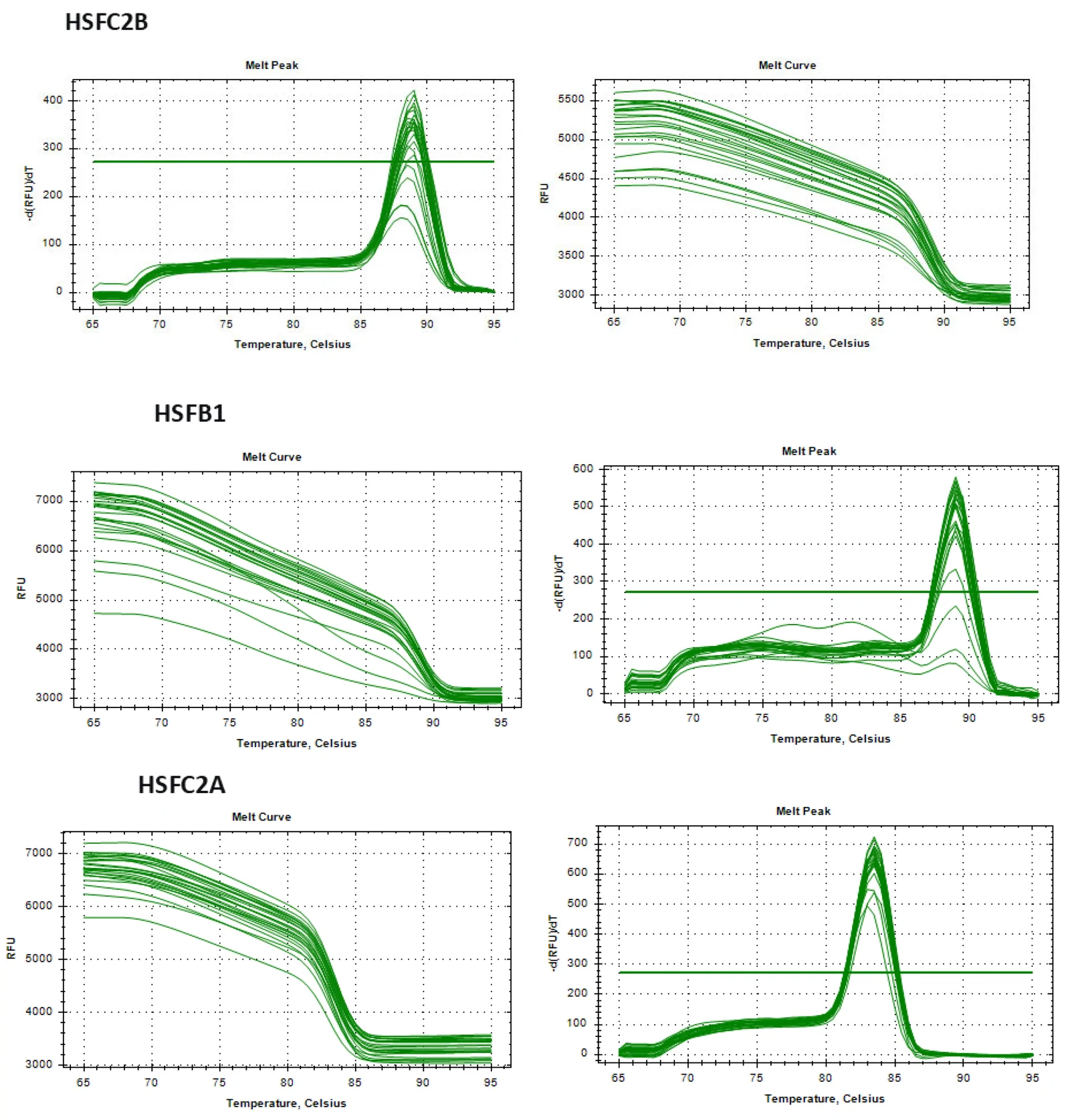
Melting curve and melting peaks of three genes analyzed by qRT-PCR.

The TRIzol technique was employed to extract total RNA from the treated seedlings. The concentration and purity of the extracted RNA were examined using a NanoDrop spectrophotometer (Thermo Fisher Scientific, Waltham, MA, USA), and RNA integrity was verified by 1.0% agarose gel electrophoresis (Vazyme, Wuhan, China) [73]. The reverse transcriptase kit PrimeScriptTM RT (Vazyme, Wuhan, China) Reagent Kit with gDNA Eraser was employed for the synthesis of cDNA. The obtained cDNA was diluted to a final concentration of 100 ng/µL and preserved at −20 °C for qRT-PCR. Primers were prepared using Primer3 Plus software and validated using Oligo Analyzer (IDT) (https://www.idtdna.com/pages/tools/oligoanalyzer) (accessed on 20 November 2025) (Table A1). A LightCycler^®^ 480 II Real-Time PCR System (Bio Rad, 11000 Touch, Singapore) with TB Green^®^ Premix Ex Taq™ II was used to perform qPCR. The reaction mixture (20 µL) contained 10 µL of SYBR Green Master Mix (Vazyme, Wuhan, China), 0.4 µM each of forward and reverse primers, and 2 µL of diluted cDNA. The thermal cycling process was as follows: 95 °C for 30 s (initial denaturation), followed by 40 rounds of 95 °C for 5 s and 60 °C for 30 s. The comparative expression levels of target genes were measured using the 2^−^ΔΔCt method [72], with OsActin (Tsingke, Wuhan, China) as the internal reference gene due to its more stable expression compared with other internal controls [74,75]. Three biological replicates and three technical replicates were prepared for each sample. The melting peak and melting curves are provided in the Appendix A (Figure A1).

### 3.10. Statistical Analyses

Relative expression was calculated and presented as mean ± standard deviation (biological replicates, n = 3). Two-way ANOVA (*p* < 0.05) was performed using RStudio software (v2025.05.1) to identify the statistical significance difference between cultivars, time points, and cultivars x time points interactions for three genes.

## 4. Conclusions

In this study, we identified a total of 25 *OsHSFs* in indica and japonica cultivars through a comprehensive genome-wide analysis of rice. The findings indicated that *OsHSFs* are involved in the HS-response pathway, which is activated by heat stress. In silico comparative analysis indicated cultivar-specific differences in the expression of *OsHSF* genes between indica and japonica cultivar under HS. Furthermore, the expression profile of three candidate genes, *HSFB1*, *HSFC2A*, and *HSFC2B*, demonstrated that such genes play a major role in HS responses. At the 6 h point, the Nipponbare cultivar showed significantly higher expression of *HSFC2A*, *HSFB1*, *and HSFC2B* compared to the 9311 cultivar. Furthermore, the *HSFC2B* gene not only showed significant differences for individual cultivars and time point comparisons, but it also showed significant statistical differences for cultivar × time point interactions compared to the other two genes. These findings provide potential targets for breeders to explore, particularly based on *OsHSFs* like *HSFC2B* in the japonica cultivar. However, there is a need to conduct comprehensive RNA profiling to find potential candidate *OsHSFs* genes and other regulatory mechanisms controlling heat resilience in rice. Future research is also required to validate the functions of candidate genes, particularly through knockout methods using the CRISPR/Cas-9 system. Such research will provide a deeper understanding of the role of *OsHSFs* in alleviating HS and developing tolerant cultivars. Nevertheless, the present study provided useful information for stakeholders and also ensured commitment towards Sustainable Development Goals (SDGs): SDG#2 (Zero Hunger) and SDG#13 (Climate Action).

## Figures and Tables

**Table 1:** Comprehensive information regarding *OsHSF* family including accession number, gene name, and functions.

Sr. No.	MSU ID + Alternative Splice Forms of (*O. Japonica*)	Accession Number/RAP (Os-ID)	Gene ID (*O. Indica*)	Gene Name	Genomic Length	CDS Length	Protein Length	Protein Name	Putative Function
1	*LOC_Os01g39020.1*	Os01g0571300	BGIOSGA003800	*HSFA7*	2975 nucleotides	1209 nucleotides	402 amino acids	HS transcription factor *A7*	*HSF*-type DNA-binding domain-containing protein, expressed
2	*LOC_Os01g43590.1* *LOC_Os01g43590.2*	Os01g0625300	N/A	*HSFC1A*	1650 nucleotides	1047 nucleotides	348 amino acids	HS transcription factor *C1A*	*HSF*-type DNA-binding domain-containing protein, expressed
3	*LOC_Os01g53220.1*	Os01g0733200	BGIOSGA004402	*HSFC1B*	1259 nucleotides	753 nucleotides	250 amino acids	HS transcription factor *C1B*	*HSF*-type DNA-binding domain-containing protein, expressed
4	*LOC_Os01g54550.1*	Os01g0749300	BGIOSGA004459	*HSFA4A*	2535 nucleotides	1323 nucleotides	440 amino acids	HS transcription factor *A4A*	*HSF*-type DNA-binding domain-containing protein, expressed
5	*LOC_Os02g13800.1*	Os02g0232000	BGIOSGA007838	*HSFC2A*	1575 nucleotides	897 nucleotides	298 amino acids	HS transcription factor *C2A*	*HSF*-type DNA-binding domain-containing protein, expressed
6	*LOC_Os02g29340.1*	Os02g0496100	BGIOSGA006438	*HSFA5*	3347 nucleotides	1428 nucleotides	475 amino acids	HS transcription factor *HSFA5*	*HSF*-type DNA-binding domain-containing protein, expressed
7	*LOC_Os02g32590.1* *LOC_Os02g32590.2*	Os02g0527300	BGIOSGA006348	*HSFA3*	3464 nucleotides	1497 nucleotides	498 amino acids	HS transcription factor *HSFA3*	*HSF*-type DNA-binding domain-containing protein, expressed
8	*LOC_Os03g06630.1* *LOC_Os03g06630.2*	Os03g0161900	BGIOSGA011916	*HSFA2D*	2295 nucleotides	1140 nucleotides	379 amino acids	HS transcription factor *HSFA2D*	HS transcription factor, putative, expressed
9	*LOC_Os03g12370.1* *LOC_Os03g12370.4* *LOC_Os03g12370.3* *LOC_Os03g12370.2*	Os03g0224700	BGIOSGA012151	*HSFA9*	3832 nucleotides	1233 nucleotides	410 amino acids	HS transcription factor *HSFA9*	*HSF*-type DNA-binding domain-containing protein, expressed
10	*LOC_Os03g25080.1*	Os03g0366800	BGIOSGA010674	*HSFB4D*	1907 nucleotides	1500 nucleotides	499 amino acids	HS transcription factor *HSFB4D*	F-Box domain-containing protein, expressed
11	*LOC_Os03g53340.2* *LOC_Os03g53340.6* *LOC_Os03g53340.5* *LOC_Os03g53340.4* *LOC_Os03g53340.3* *LOC_Os03g53340.1*	Os03g0745000	BGIOSGA009826	*HSFA2A*	3201 nucleotides	1131 nucleotides	376 amino acids	HS transcription factor *HSFA2A*	*HSF*-type DNA-binding domain-containing protein, expressed
12	*LOC_Os03g58160.1* *LOC_Os03g58160.2*	Os03g0795900	BGIOSGA013724	*HSFA2E*	3264 nucleotides	1074 nucleotides	357 amino acids	HS transcription factor *HSFA2E*	HS transcription factor, putative, expressed
13	*LOC_Os03g63750.1*	Os03g0854500	BGIOSGA013971	*HSFA1*	3625 nucleotides	1521 nucleotides	506 amino acids	HS transcription factor *HSFA1*	*HSF*-type DNA-binding domain-containing protein, expressed
14	*LOC_Os04g48030.1*	Os04g0568700	BGIOSGA016941	*HSFB2A*	1838 nucleotides	918 nucleotides	305 amino acids	HS transcription factor *HSFB2A*	HS transcription factor *B-1* putative, expressed
15	*LOC_Os05g45410.1*	Os05g0530400	BGIOSGA020240	*HSFA4D*	2476 nucleotides	1380 nucleotides	459 amino acids	HS transcription factor *HSFA4D*	*HSF*-type DNA-binding domain-containing protein, expressed
16	*LOC_Os06g35960.1*	Os06g0553100	BGIOSGA021054	*HSFC2B*	2406 nucleotides	837 nucleotides	278 amino acids	HS transcription factor *HSFC2B*	*HSF*-type DNA-binding domain-containing protein, expressed
17	*LOC_Os06g36930.1*	Os06g0565200	BGIOSGA023100	*HSFA6*	1118 nucleotides	996 nucleotides	331 amino acids	HS transcription factor *HSFA6*	*HSF*-type DNA-binding domain-containing protein, expressed
18	*LOC_Os07g08140.1*	Os07g0178600	BGIOSGA025247	*HSFA2B*	3290 nucleotides	1119 nucleotides	372 amino acids	HS transcription factor *HSFA2B*	HS transcription factor, putative, expressed
19	*LOC_Os07g44690.1*	Os07g0640900	BGIOSGA026239	*HSFB4B*	3294 nucleotides	933 nucleotides	310 amino acids	HS transcription factor *HSFB4B*	*HSF*-type DNA-binding domain-containing protein, expressed
20	*LOC_Os08g36700.1*	Os08g0471000	BGIOSGA026829	*HSFB4A*	1236 nucleotides	1143 nucleotides	380 amino acids	HS transcription factor *HSFB4A*	*HSF*-type DNA-binding domain-containing protein, expressed
21	*LOC_Os08g43334.1* *LOC_Os08g43334.2*	Os08g0546800	BGIOSGA026537	*HSFB2B*	9575 nucleotides	1851 nucleotides	616 amino acids	HS transcription factor *HSFB2B*	*HSF*-type DNA-binding domain-containing protein, expressed
22	*LOC_Os09g28200.1*	Os09g0455200	BGIOSGA029640	*HSFB4C*	1789 nucleotides	1185 nucleotides	394 amino acids	HS transcription factor *HSFB4C*	*HSF*-type DNA-binding domain-containing protein, expressed
23	*LOC_Os09g28354.1*	Os09g0456800	N/A	*HSFB1*	7536 nucleotides	909 nucleotides	302 amino acids	HS transcription factor *HSFB1*	*CPuORF39*-conserved peptide *uORF* containing transcript, expressed
24	*LOC_Os09g35790.1* *LOC_Os09g35790.3* *LOC_Os09g35790.2*	Os09g0526600	BGIOSGA029403	*HSFB2C*	3914 nucleotides	1365 nucleotides	454 amino acids	HS transcription factor *HSFB2C*	Similar to Heat factor protein 3 (*HSF3*) (HS transcription factor 3) (*HSTF3*)
25	*LOC_Os10g28340.1* *LOC_Os10g28340.5* *LOC_Os10g28340.3* *LOC_Os10g28340.2*	Os10g0419300	BGIOSGA031910	*HSFA2C*	4075 nucleotides	1077 nucleotides	358 amino acids	HS transcription factor *HSFA2C*	Similar to HS transcription factor 31 (Fragment)

**Table 2:** Chromosomal location and description of *OsHSF* family genes.

Sr. No	MSU ID	Chr. No	Strand	Start Position	End Position	Chromosome Sequence Length	Description
1.	*LOC_Os01g39020*	1	Forward	21,938,865	21,941,839	43,266,274	Similar to HS transcription factor 31 (Fragment)
2.	*LOC_Os01g43590.1* *LOC_Os01g43590.2*	1	Forward	31,370,225 24,967,398	31,372,759 24,969,047	43,200,000	Similar to HS transcription factor 31 (Fragment)
3.	*LOC_Os01g53220*	1	Forward	30,582,485	30,583,743	43,270,000	Similar to HS transcription factor 29 (Fragment)
4.	*LOC_Os01g54550*	1	Forward	31,370,225	31,372,759	43,270,000	Similar to HS transcription factor 29 (Fragment)
5.	*LOC_Os02g13800*	2	Forward	7,463,932	7,465,506	35,900,000	Similar to HS transcription factor 29 (Fragment)
6.	*LOC_Os02g29340*	2	Forward	17,428,027	17,431,373	35,900,000	Winged helix repressor DNA-binding domain-containing protein
7.	*LOC_Os02g32590*	2	Forward	19,313,057	19,309,594	35,900,000	Similar to HS transcription factor 31 (Fragment)
8.	*LOC_Os03g06630*	3	Forward	3,342,254	3,344,548	37,399,924	Similar to HS transcription factor 1 (Fragment)
9.	*LOC_Os03g12370*	3	Forward	6,537,569	6,541,400	37,399,924	Similar to HSP protein (Fragment)
10.	*LOC_Os03g25080*	3	Reverse	14,345,575	14,342,679	37,399,924	Cyclin-like F-box domain-containing protein
11.	*LOC_Os03g53340*	3	Reverse	30,607,164	30,604,067	37,399,924	Winged helix repressor DNA-binding domain-containing protein
12.	*LOC_Os03g58160*	3	Forward	33,105,828	33,109,091	37,399,924	Similar to HS transcription factor 31 (Fragment)
13.	*LOC_Os03g63750*	3	Forward	35,989,011	35,992,635	37,399,924	Similar to HS transcription factor 31 (Fragment)
14.	*LOC_Os04g48030*	4	Forward	28,574,411	28,576,248	36,078,568	Similar to HS transcription factor *Sp17* (HS transcription factor) (HS factor *RHSF10*)
15.	*LOC_Os05g45410*	5	Forward	26,344,414	26,346,889	36,400,000	Similar to HS transcription factor *Sp17* (*HSF*) (HS factor *RHSF10*)
16.	*LOC_Os06g35960*	6	Reverse	20,998,867	20,996,264	35,900,000	*HSF*-type, DNA-binding domain-containing protein
17.	*LOC_Os06g36930*	6	Forward	21,761,304	21,762,421	35,900,000	Winged helix repressor DNA-binding domain-containing protein
18.	*LOC_Os07g08140*	7	Forward	4,139,160	4,142,449	29,100,000	Similar to HS transcription factor 29 (Fragment)
19.	*LOC_Os07g44690*	7	Forward	26,673,639	26,676,932	29,100,000	Similar to HS transcription factor 33 (Fragment)
20.	*LOC_Os08g36700*	8	Reverse	23,159,531	23,158,296	28,400,000	Similar to HS factor
21.	*LOC_Os08g43334*	8	Reverse	27,390,339	27,380,765	28,400,000	Similar to HS transcription factor 33 (Fragment)
22.	*LOC_Os09g28200*	9	Reverse	17,111,077	17,109,289	22,400,000	Winged helix repressor DNA-binding domain-containing protein
23.	*LOC_Os09g28354*	9	Forward	17,221,426	17,228,961	22,400,000	Similar to HS transcription factor *Sp17* (HS transcription factor) (HS factor *RHSF10*)
24.	*LOC_Os09g35790*	9	Reverse	20,595,143	20,591,230	22,400,000	Similar to Heat factor protein 3 (*HSF3*) (HS transcription factor 3) (*HSTF3*)
25.	*LOC_Os10g28340*	10	Reverse	14,750,175	14,746,101	23,014,639	Similar to HS transcription factor 31 (Fragment)

## Data Availability

The data will be made available upon request.

## Appendix A

**Table A1:** Basic information of primers.

Primer Name	Primer Sequence
*HSFC2B-F*	ACGTACAGGATGGTGGAGGA
*HSFC2B-R*	ACCATAGGTGTTGAGCTGGC
*HSFB1-F*	GCACTGCAACTTCTCCTCCT
*HSFB1-R*	CGCAGGATTTGGAGGACTGA
*HSFC2A-F*	CCTCCTCGACTCATCCTCCA
*HSFC2A-R*	GTGTAGAGGCCGGTGAAGTC
Actin-F	GGAAGTACAGTGTCTGGATTGGAG
Actin-R	TCTTGGCTTAGCATTCTTGGGT

**Table A2:** Two-way ANOVA analysis based on cultivar and time points at statistical significance (*p* < 0.05).

Gene	Cultivar	Time Point	Cultivar x Time Point
*HSFC2A*	5.01 × 10^−11^	1.22 × 10^−6^	0.326
*HSFC2B*	5.83 × 10^−8^	1.28 × 10^−8^	7.71 × 10^−8^
*HSFB1*	0.183	0.327	0.283
